# Topology and biological function of enterovirus non-structural protein 2B as a member of the viroporin family

**DOI:** 10.1186/s13567-014-0087-6

**Published:** 2014-08-28

**Authors:** Da Ao, Shi-Qi Sun, Hui-Chen Guo

**Affiliations:** State Key Laboratory of Veterinary Etiological Biology and OIE/National Foot and Mouth Disease Reference Laboratory, Lanzhou Veterinary Research Institute, Chinese Academy of Agricultural Sciences, Xujiaping 1, Lanzhou, Gansu 730046 The People’s Republic of China; College of Veterinary Medicine, Sichuan Agricultural University, Ya’an, 625014 The People’s Republic of China

## Abstract

Viroporins are a group of transmembrane proteins with low molecular weight that are encoded by many animal viruses. Generally, viroporins are composed of 50–120 amino acid residues and possess a minimum of one hydrophobic region that interacts with the lipid bilayer and leads to dispersion. Viroporins are involved in destroying the morphology of host cells and disturbing their biological functions to complete the life cycle of the virus. The 2B proteins encoded by enteroviruses, which belong to the family *Picornaviridae*, can form transmembrane pores by oligomerization, increase the permeability of plasma membranes, disturb the homeostasis of calcium in cells, induce apoptosis, and cause autophagy; these abilities are shared among viroporins. The present paper introduces the structure and biological characteristics of various 2B proteins encoded by enteroviruses of the family *Picornaviridae* and may provide a novel idea for developing antiviral drugs.

## Table of contents

1. Introduction
2. Translation of enterovirus 2B protein
3. Transmembrane domain and membrane topology of enterovirus 2B protein
4. Effects of the ion channel characteristics of enterovirus non-structural protein 2B on host cells
  4.1 Increases the permeability of membrane structure in host cells
  4.2 Disturbs Ca^2+^ balance in host cells
  4.3 Induces apoptosis of host cells
  4.4 Induces cell autophagy
5. Strategy of antiviral drug design based on viroporins and other viral proteins with viroporin-like activity
6. Conclusion and future perspectives
7. List of abbreviations
8. Competing interests
9. Authors’ contributions
10. Authors’ information
11. Acknowledgements
12. References

## 1. Introduction

As a consequence of long-term evolution, animal viruses have acquired the ability to damage the normal physiological functions of their host cells after infection to ensure their replication and the completion of their life cycles. One of the damages inflicted is increased permeability of the host cell membrane, a phenomenon induced by the protein products of various viral genes, including viral proteases such as non-structural protein 2B and non-structural protein 3 of encephalitis B virus, glycoproteins, and viroporins [1-4].

Viroporins are a family of low-molecular weight hydrophobic transmembrane proteins encoded by animal viruses. Viroporins generally comprise 50–120 amino acid residues and have a minimum of one transmembrane domain. The transmembrane hydrophobic regions can interact with the phospholipid bilayer to induce dispersion, thus increasing membrane permeability and promoting the release of viral particles [2-9]. With the increasing comprehensive studies on viroporins, their functions in the life cycle of a virus have gained significant attention. Currently, viroporins are important in promoting virus replication and in disrupting the electrochemical equilibrium in host cells. The oligomerization of monomers in the membrane structure of host cells leads to the formation of hydrophobic pores, which increase the permeability of the host cell membrane and promote the budding of viral particles. Moreover, viroporins affect the cell vesicular transport system and glycoprotein transport process. In addition, other studies have demonstrated that viroporins are involved in destroying the shape of host cells and in disturbing their normal physiological functions to ensure completion of the entire viral life cycle. Virologists are currently focusing on the biological functions of viroporins and their involvement in viral pathogenesis.

A study in 1992 showed that influenza A virus M2 protein exhibits viroporin activity and has important functions in the viral life cycle [10]. Subsequently, proteins encoded by other viruses, such as the non-structural protein 2B of enterovirus, have similar characteristics [7]. In this review, we summarize the virus infection mechanisms by introducing the topology of enterovirus non-structural protein 2B and its biological features as a viroporin to provide new ideas for studying and developing novel antiviral intervention methods and drugs.

## 2. Translation of enterovirus 2B protein

**Figure 1:**
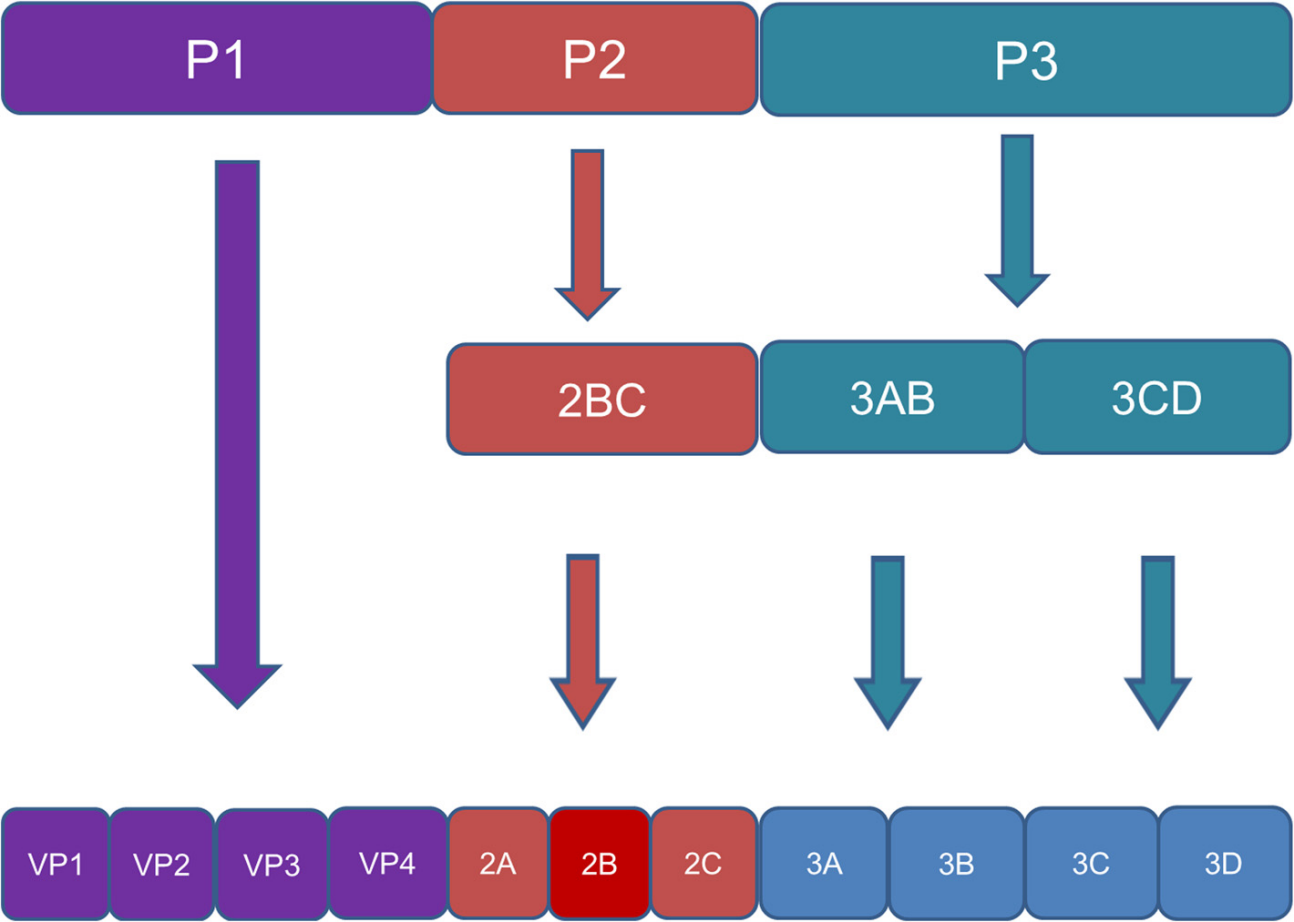
**Map of proteins encoded by the RNA of** ***Enterovirus*** **.** The *Enterovirus*-code peptide chain is factitiously divided into three segments: P1, P2, and P3. Peptide P1 is digested into four structural proteins: VP1–4, whereas peptides P2 and P3 are further digested into various non-structural proteins.

The *Enterovirus* genus belongs to the *Picornaviridae* family, which includes poliovirus, Coxsackie virus, enteric cytopathogenic human orphan virus, and human enterovirus 71 (EV71). Non-enveloped enterovirus is spherical in shape, with a diameter ranging from 28 nm to 30 nm. Its nucleocapsid is an icosahedron, and the viral capsid consists of 60 copies of capsid proteins arranged into 12 pentamers. The genome of this virus family contains a single-stranded positive-sense RNA with 7500 to 8000 nucleotides and a single open reading frame. RNA encodes a peptide chain with approximately 2200 amino acid residues. This peptide chain is divided factitiously into three regions, P1, P2, and P3, based on the differences in its structure and function. P1 constitutes the capsid protein fraction of the virus, whereas P2 and P3 comprise the non-structural proteins of the virus. P1 is cleaved by the viral protease L^pro^ into four viral capsid proteins: 1A (VP4), 1B (VP2), 1C (VP3), and 1D (VP1). The four structural proteins envelop the viral RNA into the icosahedral capsid using a highly ordered assembly process. The peptides in P2 and P3 initially generate three non-structured protein precursors: 2BC, 3AB, and 3CD. Finally, these precursors are cleaved into several non-structural proteins by the following proteases (2A^pro^ and 3C^pro^): 2A, 2B, 2C, 3A, 3B (VPg), 3C, and 3D proteins (Figure 1) [11]. The 2B protein is one of the products from the polypeptides in the P2 region.

## 3. Transmembrane domain and membrane topology of enterovirus 2B protein

**Figure 2:**
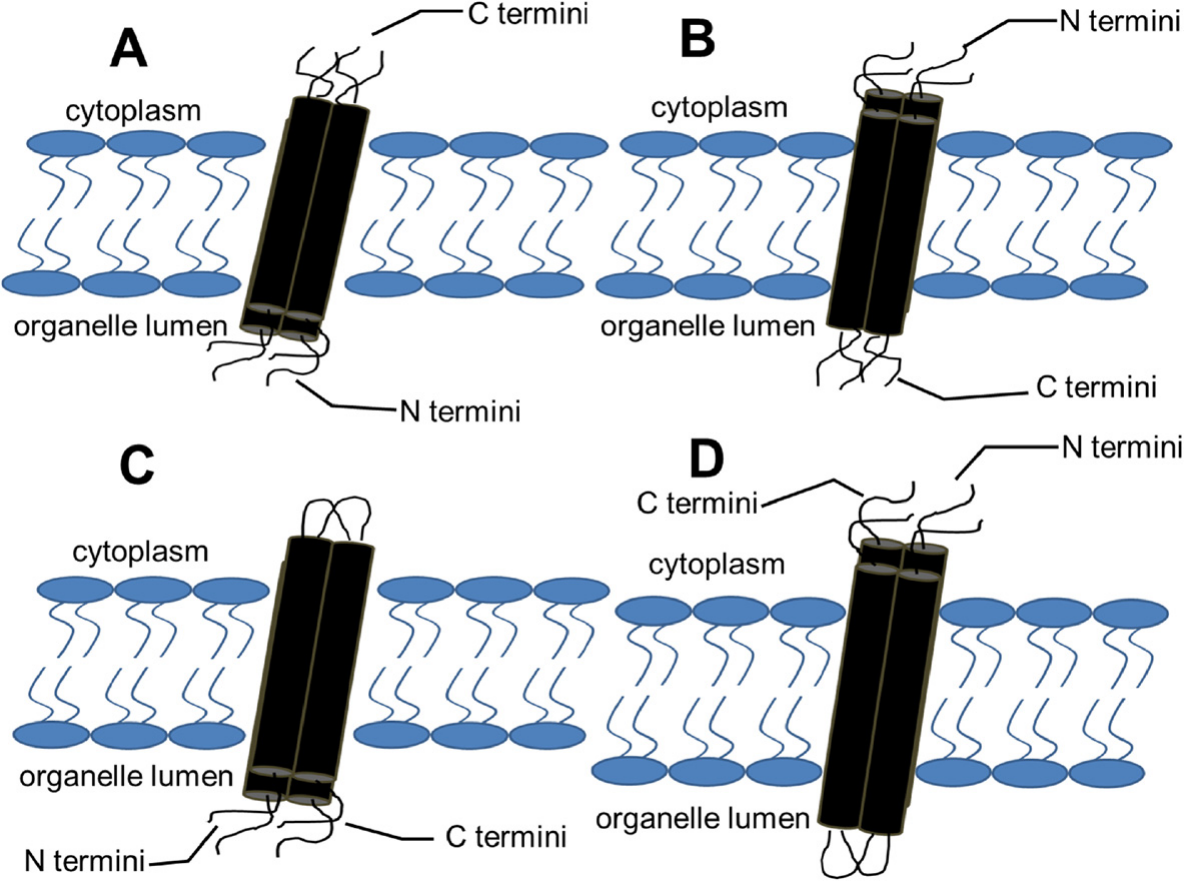
**Sketch map of the transmembrane topology of viroporins.** The four types of viroporins based on their transmembrane topologies. **(A)** and **(B)** represent type IA and type IB viroporins, respectively. These viroporins possess only one transmembrane domain with inverse orientations. **(C)** and **(D)** represent type IIA and type IIB viroporins, respectively. These viroporins possess two transmembrane domains with opposite orientations. Adapted from [12].

Various studies have provided profound insights into the viroporin structure. The currently accepted mode of action for viroporin is the polymerization of monomers to form hydrophilic pores and to exert their functions after being embedded into the host cell membrane. Viroporins can be divided into type I and type II based on the presence of one or two transmembrane regions. Moreover, both types can be further divided into subtypes IA, IB, IIA, and IIB based on their transmembrane topology [12]. Figure 2 shows the four types of viroporins. The N terminal of type IA viroporins faces the organelle lumen, and the C terminal faces the cytoplasmic matrix (Figure 2A). By contrast, the C-terminus of type IB viroporins faces the organelle lumen and the N-terminus faces the cytoplasmic matrix (Figure 2B). The C- and N-termini of type IIA viroporins stretch to the organelle lumen (Figure 2C), whereas the C- and N-termini of type IIB viroporins extend to the cytoplasm (Figure 2D).

Poliovirus 2B protein consists of approximately 100 amino acid residues and has two hydrophobic domains: HR1 (32VTSTITEKLLKNLIKIISSLVIITG55) and HR2 (61TTTVLATLALLGCDASPWQWL81). For these two hydrophobic domains, the HR1 domain crosses the cell membrane via a cationic amphiphatic α-helix, whereas the HR2 domain is a complete hydrophobic transmembrane region [13]. The HR1 and HR2 domains of the protein are located in the endoplasmic reticulum (ER) through an angled annular serial connection by mutual cooperation, such that the C- and N-termini of the polypeptide chain stretch to the ER and Golgi lumen [13-15].

The Coxsackie virus-coded 2B protein is also a transmembrane protein with 99 amino acid residues and consists of two hydrophobic domains: HR1 (37SILEKSLKALVKIISALV54) and HR2 (63LITVTATLALIGCTSSPW80) [15,16]. HR1 forms a cationic amphiphilic α-helix, whereas HR2 forms a completely hydrophobic helix [17].

Mutation analysis reveals that the lack of HR1 or HR2 causes the 2B protein to remain integrated with the host cell membrane; however, translocation across the membrane or oligomerization cannot be completed. The mutant 2B protein without HR1 or HR2 has no biological activity compared with the wild-type 2B protein [13,15]. These studies have demonstrated that the HR1 and HR2 domains are necessary for the interaction of the 2B protein with the membrane structure of the host cell. Two hydrophobic domains and the stem loop connecting them interact to form a “helix-angle-helix” structure. After the interaction, the protein could successfully embed into the membrane. Finally, normal oligomerization is completed and transmembrane pores with biological function are formed [13].

The poliovirus-coded 2B protein belongs to type IIB, whereas Coxsackie virus-coded 2B protein is a member of type IIA viroporins. This classification is based on the membrane topologies and on the classification criteria mentioned.

On the basis of the mutation of hydrophobic domains of the enterovirus-coded 2B protein, we speculate that the integrality of these hydrophobic structures and the interactions between them are crucial to the biological functions of the 2B protein. These findings may provide a breakthrough for future research and development of antiviral drugs.

## 4. Effects of the ion channel activity of enterovirus 2B protein on host cells

After infecting susceptible cells with an animal virus, the structure and function of the host cells significantly change. These changes include increased intracellular vesicle formation, increased membrane permeability, disrupted Ca^2+^ balance in cells, cell apoptosis, and induced autophagy [5,13]. Some of these functional and morphological changes can also be observed in cells expressing the enterovirus-coded 2B protein only [9,13,18,19]. The viroporin features of the 2B protein that affect the biological functions of host cells are summarized in the following aspects.

### 4.1 Increases the permeability of the membrane structure in host cells

Biochemical analysis of the enterovirus 2B protein sequence shows its two hydrophobic domains [15]. Self-polymerization reaction in a single lipid membrane in vitro, in vivo yeast and mammalian cell two-hybrid analysis, and fluorescence resonance energy transfer microscopy of live cells confirm that the 2B protein is a transmembrane protein [14,20]. Moreover, data obtained from the biochemical analysis of the 2B protein are highly consistent with its molecular dynamic data. Previous studies have shown that the C- and N-termini of poliovirus 2B protein are exposed to the ER and Golgi lumen when expressed in cells [13-15]; however, the C and N-termini of Coxsackie virus 2B protein are located in the cytoplasm [15,16]. The 2B protein may assemble into hydrophilic pores with diameters ranging from 5 Å to 7 Å in the form of a tetramer in the lipid bilayer [21]. When mutations are induced in the hydrophilic domain (58RNHDD62) between HR1 and HR2 of Coxsackie virus 2B protein, the protein cannot self-polymerize in the lipid layer in vitro or in the host cell membrane [14,22].

After being infected with an animal virus, a typical pathological change of the host cells is the significant increase in the permeability of the cellular membrane. The increased permeability starts from the changes in Ca^2+^ content and the disruption of ionic gradients outside the membrane [18]. When the enterovirus-coded 2B protein is expressed independently in susceptible cells, cell permeability increases, such that non-specific selective ions and solutes with low molecular weights (< 1000 Da) freely flow in or out [12,13]. The increased membrane permeability in the cells infected with enteroviruses or the cells overexpressing the 2B protein has been demonstrated by the following experiments. The β-galactosidase in *Escherichia coli* cannot be released and O-nitrophenyl β-D-galactopyranoside (ONPG) cannot pass into *E. coli* through the complete cell membrane under normal conditions. However, after inducing the expression of the 2B protein in *E. coli* BL21 (DE3), the addition of ONPG to the bacterial culture suspension changes the color to yellow [23]; its interaction with β-galactosidase forms a yellow product. Second, the impermeable translation inhibitor hygromycin B was used to reveal that the virus-coded 2B protein can increase the permeability of the membrane [24]. Furthermore, non-specific selective ions and solutes with low molecular weights (< 1500 Da) freely pass through the plasma membrane when poliovirus 2B protein is embedded into the liposomes or expressed in BHK-21 cells; moreover, the intrinsic proton gradients at both sides of the membrane are disturbed [9,13]. Similar to the function of poliovirus 2B protein, membrane permeability increases after the 2B gene in Coxsackie virus B3 was expressed in green monkey kidney cells [25]. Except when proton gradients at both sides are destroyed and when hygromycin B is allowed to freely flow outside, the pH in the Golgi membrane evidently increases with the development of infection [19]. All these studies have proven that the 2B protein increases the permeability of the host cell plasma membrane.

The following two mechanisms are initially hypothesized to be responsible for the increase in cell membrane permeability: (1) after polypeptide chain cleavage, the resulting mature protein covers the membrane surface in a carpet pattern to destroy the bending degree of the phospholipid bilayer, thereby affecting membrane permeability; and (2) after polypeptide chain cleavage, the monomers of the protein are formed. The oligomerization in the membrane structure of host cells and the formation of transmembrane pores subsequently occur to increase membrane permeability [26]. The increased permeability causes damage in the membrane structure of host cells. This process is very important for the virus to complete its life cycle. The increased cell membrane permeability changes the original physiological state of the host cell and facilitates the completion of replication and viral assembly of a virus, as well as the budding of the progeny virus. The enterovirus-coded 2B protein forms transmembrane pores that rely on the two hydrophobic transmembrane domains only. This established phenomenon affects membrane permeability and has very important functions in the viral life cycle.

### 4.2 Disturbs Ca^2+^ balance in host cells

Another significant effect of enterovirus 2B protein on host cells is the disruption of Ca^2+^ balance in cells. Ca^2+^, an important ion in cells, controls the activation of various intracellular enzymes and is involved in their catalytic processes. Ca^2+^ also has important functions in viral replication and various physiological processes [27]. At normal physiological state, intracellular Ca^2+^ retains a dynamic balance in the free and bound states. After infecting the cells with poliovirus for 4 h, the Ca^2+^ content in the cytoplasm increases. The 2B precursor 2BC also exerts similar effect after being expressed in HeLa cells [18]. Some studies have reported that the independent expression of Coxsackie virus 2B protein is sufficient to increase the Ca^2+^ levels in the cytoplasm significantly [28].

Preliminary studies have shown that the Ca^2+^ levels in the cytoplasm increase in cells expressing the 2B protein only, but the source of Ca^2+^ ions is unknown. Moreover, Coxsackie virus infection induces a decrease in the Ca^2+^ content in the ER and Golgi, as well as a decrease in the mitochondrial uptake of Ca^2+^ [28,29]. On the basis of these observations, scholars have proposed two possible mechanisms of the 2B protein: (1) induces the extracellular Ca^2+^ inflow; and (2) disrupts the ER and Golgi membranes to cause outflow of the stored Ca^2+^ ions into the cytoplasm [30].

Ca^2+^–ATPase in the ER can transfer Ca^2+^ from the cytoplasm into the ER to compensate for the lost Ca^2+^. Using thapsigargin to inhibit the activation of Ca^2+^–ATPase in the ER reveals that the expressions of poliovirus and Coxsackie virus 2B proteins in host cells promote Ca^2+^ outflow from the ER [18,30]. However, the increased Ca^2+^concentration in the ER is mainly due to the extracellular Ca^2+^ inflow [18].

Some studies used poliovirus to infect human neuroblastoma IMR5 cells, which grow in a culture medium without Ca^2+^. As a result, the Ca^2+^ concentrations in the ER increase [31]. Inhibiting the ryanodine receptor (RyR) and inositol-1,4,5-triphosphate receptor (IP3R), which control the Ca^2+^ inflow and outflow in the ER, decreases the Ca^2+^ content in the cytoplasm. These results indicate that the virus affects RyR and IP3R to alter the Ca^2+^ levels [31]. However, no direct evidence has supported the conclusion that the 2B protein induces changes in the Ca^2+^ content. More complex mechanisms are possibly involved, and further detailed investigations should be carried out.

### 4.3 Regulates apoptosis in host cells

Apoptosis is a process of cell death caused by various stimuli, including virus infection. This process is achieved via the following two pathways: (1) exogenous pathway, where stimuli activate the death receptors Fas or CD95 on the cell surface; and (2) endogenous pathway, where increased permeability of the mitochondrial membrane and ER stress occurs [32]. In the mitochondrial pathway, the permeability of the mitochondrial membrane increases. Transmembrane potential is disrupted, and proapoptotic molecules are released [27].

**Figure 3:**
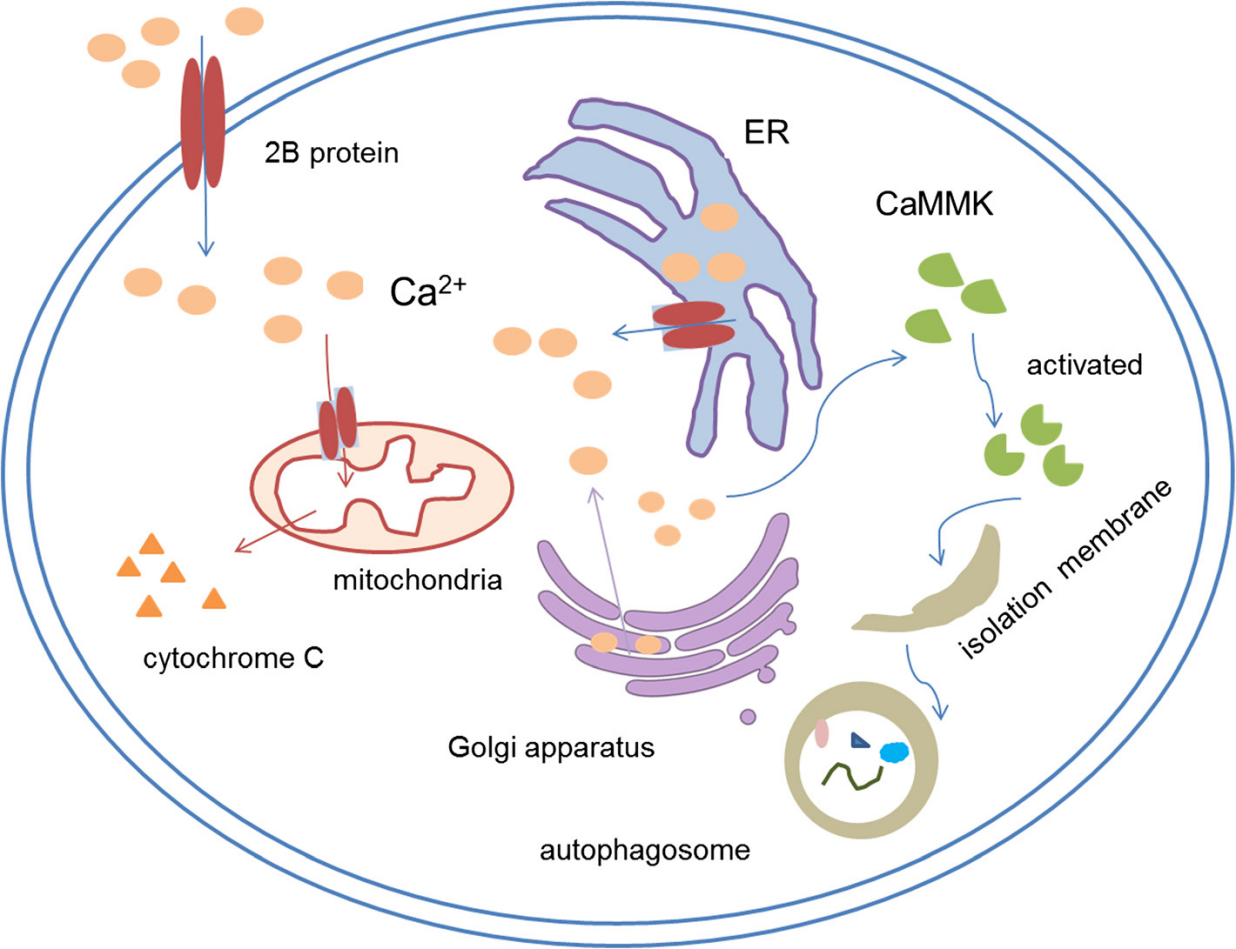
**Sketch map of apoptosis and autophagy induced by protein 2B.** The 2B protein can embed into the cytomembrane, ER, and mitochondrial membrane to increase cell permeability, disturb intracellular Ca^2+^ equilibrium, and ultimately induce apoptosis and autophagy of host cells. Adapted from [26] and [34].

An increasing number of studies have shown that the Ca^2+^ exchange between the ER and mitochondria has a very important function in the apoptotic process in the ER-dependent pathway [27-29,33]. The excessive mitochondrial uptake of Ca^2+^ has toxic effects on cells; high Ca^2+^ concentrations can open the mitochondrial permeability transition pores, expand mitochondrial permeability, and rupture the mitochondrial outer membrane. Consequently, cytochrome C and other pro-apoptotic factors are released, eventually leading to apoptosis (Figure 3).

Zhou et al. found that the expression of poliovirus and Coxsackie virus 2B proteins can decrease the Ca^2+^ levels in the ER, Golgi, and mitochondria [27]. De Jong et al. also found a similar effect of Coxsackie virus 2B protein on the efflux of Ca^2+^ in the Golgi apparatus [19]. This effect possibly has two functions. The first function is the inhibition of the protein transition pathway in cells to promote viral replication and repression of the antiviral immune response of host cells. The decreased Ca^2+^ content in these organelles inhibits the transport of protein by the vesicles. The vesicles generated from the ER or Golgi also accumulate in the cytoplasm. Viral RNA replication occurs in the vesicles [27]. All viral proteins required for RNA replication and the newly synthesized viral RNA are associated with the surfaces of these virus-induced vesicles. The cells infected with poliovirus have many accumulated virus-induced vesicles. After isolation from infected cells, the replication complex is found in the center of a rosette-like cluster formed by many virus-induced vesicles [35,36]. The replication complex is attached to the vesicular membranes and is enclosed through the plus strands of the replicative intermediate protrude from the central part of the complex. Finally, progeny 36S RNA is released on the surface of the rosette [36]. In addition, the alphavirus RNA replication complexes on the surfaces of the membranous vesicles have endosomal origin [37]. The formation of the replication complexes requires active viral RNA replication. The membrane structure is essential for initiating RNA synthesis reaction [36-38]. The second function is temporary anti-apoptosis. After viral invasion, the apoptotic program immediately starts in the cells. However, at the middle stages of viral infection, apoptosis suddenly stops. Mature apoptosis subsequently recovers at the late stages of viral infection.

Poliovirus and Coxsackie virus can initiate the apoptotic program in HeLa cells after infection through inhibiting intracellular transcription. However, at the start of infection, the apoptosis of host cells suddenly stops with the development of viral replication [29]. After viral replication, the entire apoptotic signal becomes evident [39]. These results demonstrate that apoptosis is partially inhibited or delayed to ensure that viral replication processes are completed after enterovirus infection.

Poly-(ADP-ribose) polymerase, a caspase-3 substrate, is cleaved significantly in the poliovirus 2B protein-expressing cells, and caspase-3 is activated subsequently. The mitochondria in these cells expressing poliovirus 2B protein exhibit punctated entities instead of the typical long shape [5]. These results reveal that poliovirus 2B protein can induce apoptosis. Moreover, the Coxsackie virus 2B protein expressed in HeLa cells inhibits apoptosis caused by cycloheximide and actinomycin D [28]. An evidence also indicate that these phenomena are due to the disruption of Ca^2+^ balance in cells caused by the 2B protein [29]. Moreover, the 2B protein forms transmembrane pores in the ER and Golgi membrane; thereby, decreasing the Ca^2+^ content and generating extracellular Ca^2+^ inflow. In addition, the uptake of Ca^2+^ by the mitochondrion decreases and the Ca^2+^ signal transmission between the ER and mitochondrion is disrupted; thus, apoptosis is terminated. At the late stage of viral infection, the permeability or potential energy of the mitochondrial membrane is damaged and thus causes the mitochondria to absorb the excess Ca^2+^ and release very high amounts of cytochrome C [27]. The mature apoptotic signal in the host cells was observed, and a mass of cytochrome C was detected in the cytoplasm. The infected cells eventually die, and viral particles are enclosed in the apoptotic bodies whose phagocytosis by neighboring cells provide a potential method for viral distribution [40].

### 4.4 Regulates cell autophagy

The infected cells ultimately undergo apoptosis to release viral particles. However, at the start of viral infection, apoptosis does not immediately occur to contend with viral infection but autophagy occurs first. Autophagy is a necessary process to balance the degrading and reusing of the intracellular components in response to nutritional deficiency and other stresses [41,42]. Some studies have shown that cell autophagy can balance the favorable and unfavorable effects of organism immunity and inflammation to help organisms fight various contagious autoimmune and inflammatory diseases [34]. Other studies have also demonstrated that EV71 infection increases the number of autophagosomes in cells and inhibits the cell apoptotic pathway [43]. A mass of viruses are replicated during this period, which is similar to a phenomenon observed when Coxsackie virus B3 is used to infect cells [44].

Many viroporins, such as influenza A virus-encoded M2 protein, rotavirus-encoded non-structure protein 1, non-structural protein 4 (NSP4), and yellow fever virus-encoded non-structural protein 4A, can induce cell autophagy and inhibit cell apoptosis to promote viral multiplication [45-48]. Studies on NSP4 of rotavirus have demonstrated that the protein induces the release of Ca^2+^ from the ER to the cytoplasm during viral replication. Ca^2+^/calmodulin-dependent protein kinases-β kinases and 5′ adenosine monophosphate-related protein-dependent signal pathways are activated resulting in cell autophagy. However, the maturation of autophagosomes is inhibited. Autophagic vesicle membranes are used to transfer viral proteins from the ER to the corresponding replication and assembly sites [48]. The maturation of autophagosomes is inhibited during enterovirus infection, and intracellular vesicles become abundant during enterovirus 2B protein process. The vesicles secreted from the Golgi apparatus function as viral RNA replication sites [27]. Other scholars have indicated that protein secretion through the Golgi apparatus is inhibited to a certain extent [33]. Our research also found that autophagy is induced after the cells were transfected with the plasmid-encoded 2B protein of foot and mouth disease virus FMDV (unpublished data).

## 5. Strategy for antiviral drug design based on viroporins and other viral proteins with viroporin-like activity

Over the recent years, studies have focused on the structure and functions of various viral viroporins; the studies and design of antiviral drugs based on viroporins and other viral proteins with viroporin-like activity have made an important breakthrough. Currently, the development of these antiviral drugs in this pathway mainly focuses on three aspects. The first aspect is the design of fusion inhibitors. The designed drugs can inhibit the fusion of protein and cell membrane. Bosch et al. indicated that the coronavirus-coded S protein is a transmembrane protein that pertained to type I viroporin. The HR1 region of the S protein can inhibit the fusion between virus and cell membranes after mutation. The second aspect is the design of the ion channel inhibitors. These inhibitors can prevent viroporin activity to form an ion channel. The inhibitory effect of amantadine on influenza A virus M2 protein illustrates a good example. The third aspect is the design of membrane protein antibodies. The antibody that specifically affects membrane proteins should be determined to inhibit viral invasion by removing the protein or blocking membrane fusion. Studies on human immunodeficiency virus-encoded envelope protein, known as gp160, confirmed the efficiency of this method [49,50].

Four inhibitors inhibit the viroporin activity based on these strategies. First, amantadine, a widely studied compound, can inhibit the uncoating of influenza A viruses by interacting with the M2 protein [51]. Amantadine is located inside the virus M2 channel; amantadine localizes to the histidine gate and facilitates the orientation of His37 imidazole rings to lie in the close conformation. In addition, amantadine functions as a blocker when interacting with three pore-lining residues: Leu26, Ala30, and Ser31 [52,53]. Amantadine also inhibits the ion channel activity of the p7 protein of Hepatitis C Virus (HCV) [54] and the C-terminal subunit of the p13 protein (p13-C) of the GB virus B (GBV-B), which is closely related to HCV [55]. Second, the long-alkyl-chain iminosugar derivatives inhibit the HCV p7 protein to form an ion channel [56]. The results show that *N*-butyl-glucose analogue deoxynojirimycin and *N*-nonyl-galactose analogue deoxygalactonojirimycin, which contain the short-alkyl-chain, do not affect the p7 channel activity. However, the long-alkyl-chain derivatives, including *N*-nonyl-glucose analogue deoxynojirimycin, *N*-nonyl-galactose analogue deoxygalactonojirimycin, and *N*-7-oxanonyl-6-deoxy-DGJ, dose-dependently inhibit the p7 channel signals with 105, 140, and 180 μM, respectively [54,56,57]. Third, amiloride, a drug used in the treatment of hypertension and congestive heart failure, blocks the ion channel activity and enhances the virus-like particle budding caused by human immunodeficiency virus 1 (HIV-1) Vpu [58]. Moreover, hexamethylene amiloride blocks the cation-selective ion channels formed by the p7 protein of HCV [59]. Finally, a novel small molecule, BIT225 (N-[5-(1-methyl-1H-pyrazol-4-yl)-napthalene-2-carbonyl]-guanidine), has been identified as a novel inhibitor of the HCV p7 ion channel [60]. Compared with the effect of BIT225 on HIV-1 release and the Vpu lacked HIV-2 from human macrophages, scholars have found that BIT225 blocks the Vpu ion channel activity and also exhibits anti-HIV-1 activity [61].

The study on the 2B protein coded by virus from *Picornaviridae* family is relatively delayed. Moreover, the hydrophobic property and diversification of the membrane topology of the 2B protein present many problems. However, cryo-electron microscopy, Fourier-transform infrared spectroscopy, molecular dynamical analogs, and other new technologies have been developed for antiviral drug research; thus, current studies on antiviral drugs based on the 2B protein mechanism considerably progress [62]. Some studies have reported that short-hairpin RNA can affect the 2B gene of Coxsackie virus B3 and inhibit viral replication [63]. Other studies have also demonstrated that BHK cells expressing poliovirus 2B protein can increase the permeability of neighboring normal cells by establishing a “connection” between the cells [64]. All these findings may provide novel methods for studying antiviral drugs based on the 2B protein.

## 6. Conclusion and future perspectives

Previous studies demonstrated that enterovirus 2B protein has a role in pore formation, and preliminarily analyses on its effects on the viral life cycle were conducted. However, the detailed functional mechanism of the 2B protein requires further study. In particular, elucidating the relationship of viroporin function with the signaling pathway in cells and with regulatory proteins may provide novel information and basis for antiviral treatment by selectively inhibiting specific pathways.

With more studies on the mechanism of enterovirus 2B protein accompanied with the emerging technologies and rapid development in multidisciplinary studies, a quantum progress in the research and development of novel antiviral drugs that target the 2B gene or protein would be expected in the near future.

## 7. Abbreviations

NS2B-NS3: Non-structural protein 2B and non-structural protein 3
ECHO-virus: Enteric cytopathogenic human orphan virus
EV71: Enterovirus 71
NSP1: Non-structure protein 1
NSP4: Non-structural protein 4
NS4A: Non-structural protein 4A
ER: Endoplasmic reticulum
IAV: Influenza A viruses
HCV: Hepatitis C virus
p13-C: C-terminal subunit of the p13 protein
GBV-B: GB virus B
*N*B-DNJ: *N*-butyl-glucose analogue deoxynojirimycin
*N*N-DGJ: *N*-nonyl-galactose analogue deoxygalactonojirimycin
*N*N-DNJ: *N*-nonyl-glucose analogue deoxynojirimycin
*N*B-DGJ: *N*-nonyl-galactose analogue deoxygalactonojirimycin
HIV-1: Human immunodeficiency virus 1
HIV-2: Human immunodeficiency virus 2
BIT225: N-[5-(1-methyl-1H-pyrazol-4-yl)-napthalene-2-carbonyl]-guanidine
